# Effects of Carbonate Apatite and Bone Mixture on Bone and Soft Tissue Integration in a Rat Implant Model

**DOI:** 10.1111/cid.70077

**Published:** 2025-07-02

**Authors:** Tingyu Xie, Ikiru Atsuta, Ikue Narimatsu, Bin Ji, Kiyoshi Koyano, Yasunori Ayukawa

**Affiliations:** ^1^ Section of Implant and Rehabilitative Dentistry, Division of Oral Rehabilitation, Faculty of Dental Science Kyushu University Fukuoka Japan; ^2^ Division of Advanced Dental Devices and Therapeutics, Faculty of Dental Science Kyushu University Fukuoka Japan; ^3^ Section of Fixed Prosthodontics, Division of Oral Rehabilitation, Faculty of Dental Science Kyushu University Fukuoka Japan

**Keywords:** autogenous bone, carbonate apatite, osseointegration, osteoinductivity, rat maxillary implant model, titanium dental implant

## Abstract

**Background:**

Achieving stable bone regeneration and soft tissue integration is critical for the success of dental implants, especially in patients with alveolar bone defects. Carbonate apatite (CO_3_Ap), a synthetic bone substitute, has emerged as a promising alternative due to its excellent osteoconductivity and biocompatibility. However, CO_3_Ap lacks osteoinductive capacity, which limits its effectiveness in promoting bone regeneration on its own.

**Purpose:**

This study aimed to evaluate the effects of a CO_3_Ap–autogenous bone (AB) mixture on bone regeneration and soft tissue integration in a rat maxillary implant model.

**Materials and Methods:**

Sixty rats underwent extraction of their maxillary molars, followed by titanium implant placement. The extraction sockets were filled with three different materials: CO_3_Ap, AB, or a CO_3_Ap–AB mixture. In vivo bone tissue and soft tissue evaluations were performed at specified time points. Additionally, in vitro experiments were conducted to assess the osteogenic differentiation of mesenchymal stem cells when exposed to the CO_3_Ap–AB mixture.

**Results:**

In vivo experiments showed that the CO_3_Ap–AB mixture significantly enhanced bone volume and maintained high bone mineral density compared to CO3Ap and AB alone. Furthermore, the mixture promoted longer epithelial attachment, suggesting its potential for long‐term soft tissue stabilization. In vitro, the CO_3_Ap–AB mixture effectively promoted osteogenic differentiation of mesenchymal stem cells.

**Conclusions:**

The combination of CO_3_Ap and AB exhibited a synergistic effect, enhancing early bone regeneration, osseointegration, and soft tissue sealing, which are crucial for implant stability. The CO_3_Ap–AB mixture shows great potential as a clinically effective bone substitute for dental implant treatment in patients with compromised bone conditions.

## 1 Introduction

Dental implants have become a widely accepted standard for replacing missing natural teeth [1, 2]. The success of implant therapy is critically dependent on the condition of the alveolar bone, because adequate bone volume and quality are essential for achieving stable osseointegration [3, 4]. However, bone loss in the alveolar ridge due to trauma, periodontal disease, or tooth extraction frequently necessitates the use of bone substitutes during implant placement.

Autogenous bone (AB) remains the gold standard among bone grafting materials because of its superior osteoconductive, osteoinductive, and osteogenic properties [5, 6]. These characteristics enable AB to directly support bone growth, induce stem cell differentiation, and promote new bone tissue formation. However, its clinical use is limited by several challenges, including donor site morbidity, limited harvest volume, unpredictable resorption rates, and the need for additional surgical procedures. To overcome these limitations, various synthetic bone substitutes have been developed and tested [7].

An ideal artificial bone substitute should exhibit excellent biocompatibility, preventing immune reactions and facilitating integration with the host tissues. It must possess adequate mechanical strength to maintain structural stability during the bone regeneration process. Additionally, it should exhibit controlled resorption, serving as a scaffold for new bone formation while gradually being replaced by natural bone [8, 9].

Among synthetic materials, carbonate apatite (CO_3_Ap) closely resembles the inorganic component of natural bone [10]. It has displayed excellent osteoconductivity and can be resorbed by osteoclasts in the weakly acidic environment of bone remodeling sites [11, 12]. Studies have demonstrated that CO_3_Ap supports new bone formation in extraction sockets and promotes soft tissue closure [13], making it a promising alternative to AB.

Despite its advantages, CO_3_Ap lacks osteoinductive properties—the ability to stimulate stem cells to differentiate into osteoblasts—a hallmark of AB [14]. Osteoinductive potential arises from growth factors and signaling molecules naturally present in AB, even in its dried form [15]. Recent studies suggested that combining CO_3_Ap with AB may enhance its osteogenic potential. For example, in long bone defect models, the combination demonstrated improved bone regeneration compared with either CO_3_Ap or AB alone [16]. However, the oral cavity presents a unique environment characterized by higher blood flow, dynamic mechanical loading, and specific soft tissue interactions, all of which may influence the integration and healing process around implants [17].

Therefore, it remains unclear whether combining CO_3_Ap with AB can achieve superior bone and soft tissue integration in the oral cavity. This study aims to evaluate the effects of a CO_3_Ap–AB mixture on soft tissue integration and osseointegration using a rat maxillary implant model. By comparing this mixture to CO_3_Ap and AB alone, we seek to determine its potential as an effective bone substitute for clinical implant applications.

## 2 Materials and Methods

### 2.1 Materials

Three types of bone graft materials were used in this study. CO_3_Ap was obtained from Cytrans (GC, Tokyo, Japan) as a pure CO_3_Ap dense granule with a particle size ranging over 300–600 μm [10]. AB was harvested from the femur and tibia of three 6‐week‐old Wistar rats (Kyudo Co., Saga, Japan) using a bone scraper, followed by ultra‐violet sterilization for 48 h, which can retain protein activity to a greater extent [18]. These donor animals were distinct but of the same strain and age as the implant recipients. Considering ethical and technical feasibility, we used allogeneic bone from genetically identical animals as a surrogate for AB in this rat model. Equal volumes of CO_3_Ap and AB powder were combined as a bone substitute mixture for the Mix group. This 1:1 volume ratio was selected to ensure consistency in the limited defect volume available in the rat model. In future studies, we plan to investigate different mixing ratios to further optimize material performance.

To close the extraction sockets, Cytrans elashield membranes (GC) were applied. Pure titanium implants (2 mm diameter, 4.5 mm length; Sky Blue, Fukuoka, Japan) were used for implantation.

### 2.2 Animals

Sixty male Wistar rats (6 weeks old, weight 130–160 g) were used in this study (Kyudo Co.). All procedures were approved by the Institutional Animal Care and Use Committee at Kyushu University (approval number: A25‐240‐0).

### 2.3 Surgical Procedure

Extraction and implantation were performed as previously described [19]. Briefly, the first and second right maxillary molars were extracted under systemic anesthesia (0.3‐mg/kg medetomidine, 4.0‐mg/kg midazolam, and 5.0‐mg/kg butorphanol). The extraction sockets were subsequently enlarged using a ball drill with a 1.2 mm head to accommodate the implants.

Titanium implants were inserted into the prepared sockets, with the implant depth aligned to the level of the adjacent third molar, and the implant position was localized by the distobuccal root of the extracted M1 and the left lateral tooth. The sockets were subsequently filled with CO_3_Ap, AB, or a mixture of both materials in equal volume. In the control group, no filling material was applied after implant placement. A membrane was placed over the sockets, and primary closure was achieved through suturing. The rats were euthanized by overdose of systemic anesthetic at 1, 3, and 6 weeks post‐surgery for evaluation.

### 2.4 Micro‐Computed Tomography (Micro‐CT)

Maxillary specimens were collected at 6 weeks and fixed in 4% paraformaldehyde (Merck, Darmstadt, Germany) for 48 h. Micro‐CT analysis (SkyScan 1076 scanner, Bruker, Kontich, Belgium) was performed with a tube voltage of 49 kV and a current of 201 μA. Three‐dimensional software (CTAn, Bruker) was used to analyze the peri‐implant bone volume/tissue volume and bone mineral density (BMD) [20].

### 2.5 Histological Preparation and Staining

Tissue preparation was performed as previously described [11, 21]. Briefly, maxillary specimens were collected at 1, 3, and 6 weeks, fixed in 4% paraformaldehyde for 48 h, and decalcified in Kalkitox solution (Wako, Osaka, Japan) at 4°C for 24 h. All samples were photographed at the same angle and distance. The wound areas were assessed by ImageJ software. Specimens were subsequently embedded in Optimal Cutting Temperature compound (Sakura, Tokyo, Japan) and sectioned sagittally (10 μm thickness). Sections were stained with hematoxylin and eosin for bone tissue integration analysis or Ladewig staining to evaluate peri‐implant soft tissue healing and structure.

Sagittal sections were standardized based on implant profile, collected from the region fully showing the implant head and threads to where threads disappeared. Five sections were obtained per specimen at regular intervals, and the mean value was calculated as the representative measurement for each sample.

### 2.6 Mesenchymal Stem Cell (MSC) Isolation and Culture

MSCs were isolated from the femurs and tibias of rats according to a previously described protocol [22]. Briefly, bone marrow cells were flushed with essential medium, passed through a 40 μm filter, and cultured in growth medium consisting of alpha Modified Eagle Minimum Essential Medium (Thermo Fisher Scientific, Waltham, MA, US), 20% fetal bovine serum (Equitech‐Bio, Kerrville, TX, US), 2 mM L‐glutamine (Thermo Fisher Scientific), 100 U/mL penicillin (Thermo Fisher Scientific), and 55 μM 2‐mercaptoethanol (Thermo Fisher Scientific). Cell colonies that formed after 1 week of culture were passaged, and third‐generation MSCs were used for subsequent experiments.

### 2.7 Scanning Electron Microscopy (SEM)

Titanium plates were seeded with 2 × 10^4^ MSCs and cultured for 7 days in growth medium with or without materials (CO_3_Ap, AB, or mixture). Specimens were fixed with 2.5% glutaraldehyde, followed by dehydration in graded ethanol and vacuum drying. Subsequently, they were coated with an Au/Pd alloy and observed by SEM [23].

### 2.8 Proliferation Assay

MSCs were seeded onto culture dishes and titanium plates (2 × 10^4^ cells/well) and cultured for 7 days. Cell proliferation was assessed using the Cell Counting Kit‐8 (Wako) by measuring absorbance.

### 2.9 Osteogenic Differentiation

MSCs were cultured in osteogenic medium containing 10 mM L‐ascorbic acid (Wako), 200 mM β‐glycerophosphate (Merck), and 1 mM dexamethasone (Merck), with or without the materials for 7 or 14 days. Osteogenic differentiation was evaluated by determining alkaline phosphatase (ALP) activity on Days 7 and 14 and by performing Alizarin Red S staining to assess calcium deposition after 14 days [24].

### 2.10 Statistical Analysis

Data were expressed as the mean ± standard deviation. Each group consisted of five samples. Sample size was determined using the resource equation method, which calculates the error degrees of freedom (*E*) for ANOVA. The value of *E* was set between 10 and 20 to ensure adequate statistical power. As a result, five animals per group were used, accounting for possible implant loss. One‐way analysis of variance with Tukey's post hoc test was used to compare groups; Bonferroni correction was used for multiple comparisons, and a *p* value of < 0.05 was considered to be statistically significant.

## 3 Results

### 3.1 Visual Observation of Soft Tissue Healing

**FIGURE 1:**
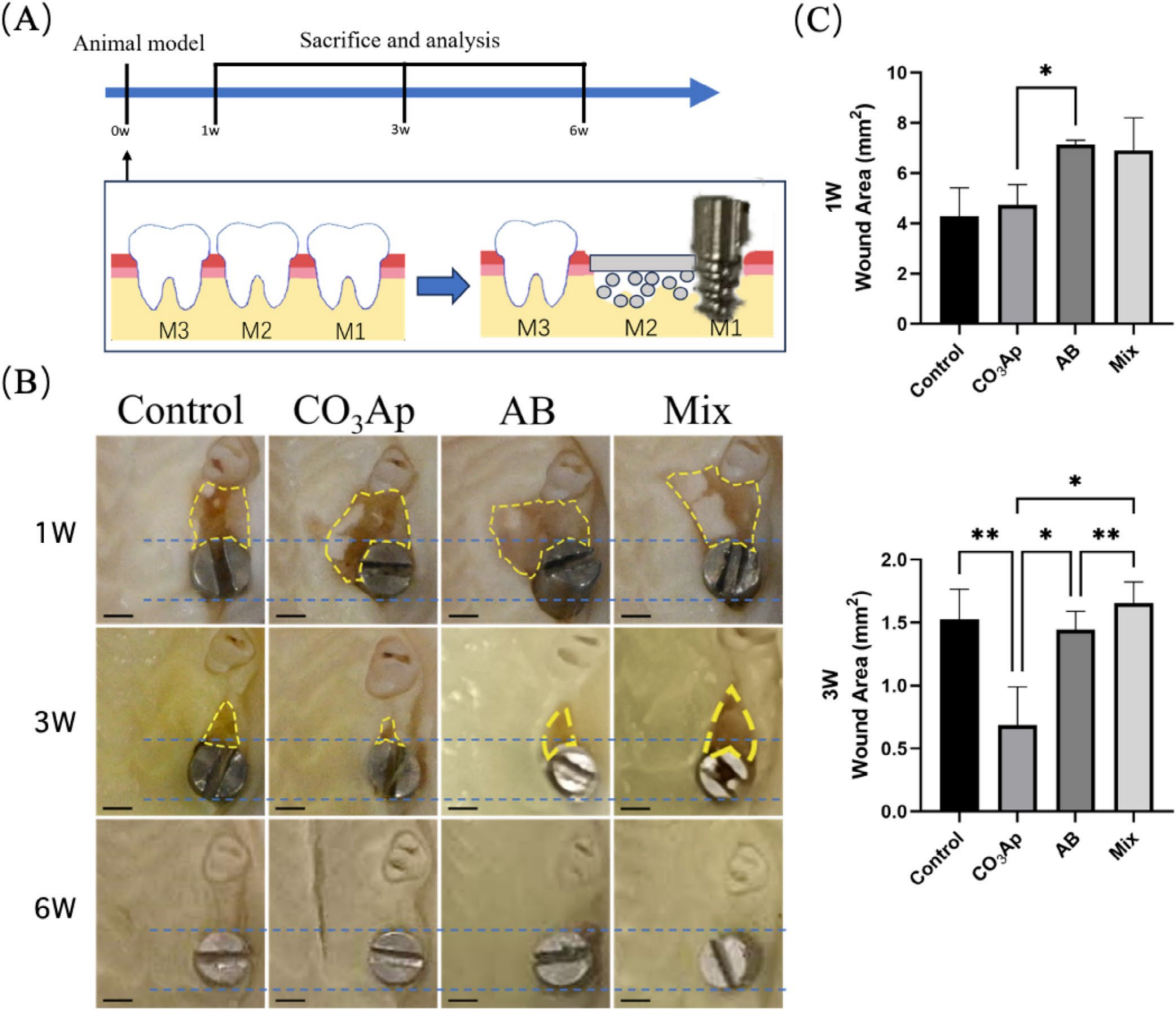
Macroscopical observation of soft tissue closure. (A) Animal model and protocol. (B) Soft tissue closure was observed macroscopically, and wound area was measured on intra‐oral images at different times after implantation. Bar = 1 mm. (C) Wound area at 1 and 3 weeks. (*n* = 5, *p < 0.05, **p < 0.01).

The experimental timeline is presented in Figure 1A. Maxillary samples were collected, and the wound areas were assessed by ImageJ software (Figure 1B,C).

At 1 week, the CO_3_Ap group exhibited a significantly smaller wound area compared with the AB group, with no significant difference between the CO_3_Ap and Mix groups. At 3 weeks, the healing process was further advanced in the CO_3_Ap group, which displayed the smallest wound area among all groups.

### 3.2 Bone Regeneration in Extraction Sockets

**FIGURE 2:**
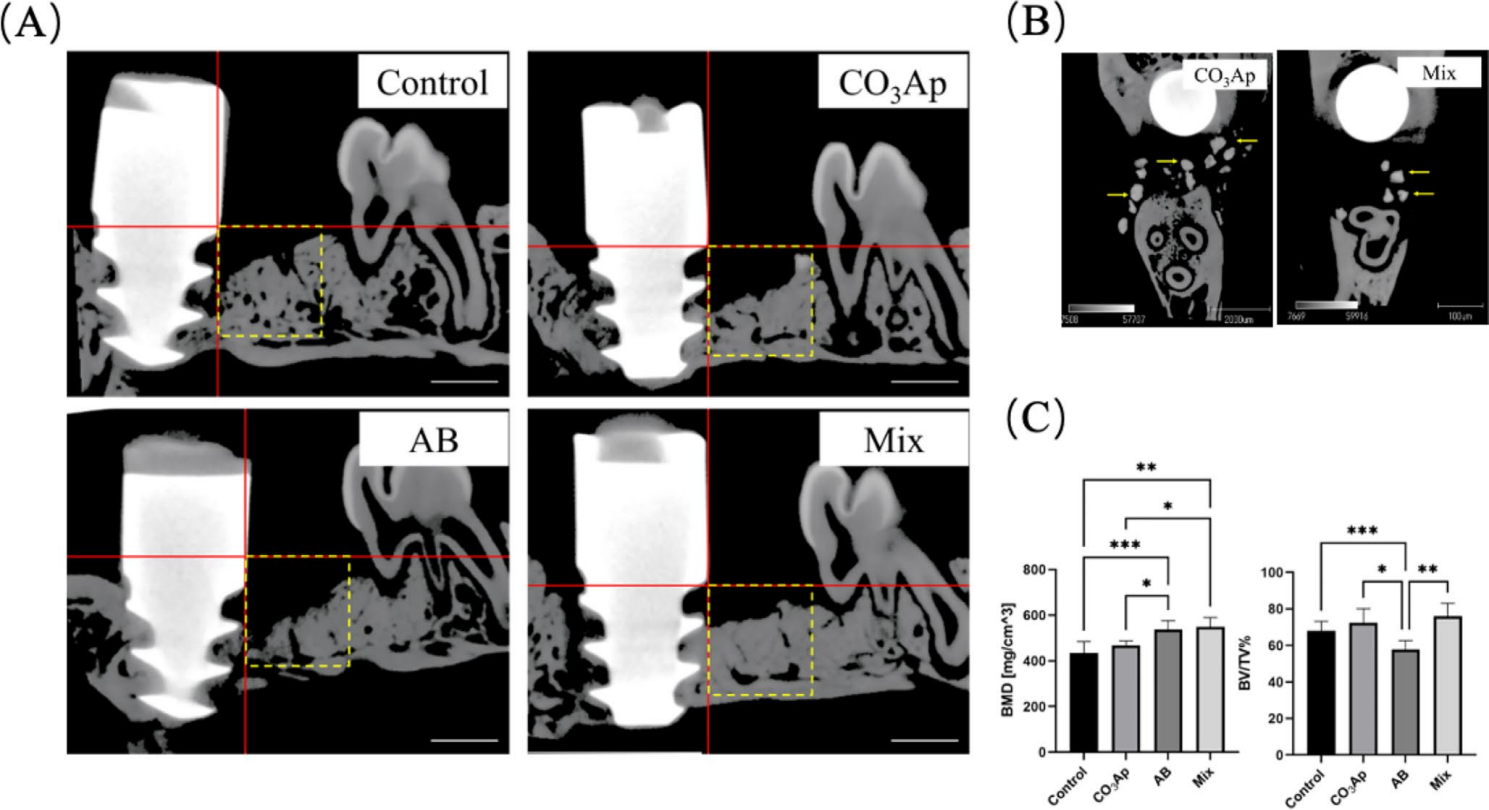
Evaluation of pre‐implant bone formation by Micro‐CT. (A) Image of sagittal section at observation site by Micro‐CT. Yellow square is the measurement area. Bar = 1 mm. (B) Bone mineral density (BMD) and bone volume/tissue volume (BV/TV) of the selected area. (*n* = 5, *p < 0.05, **p < 0.01, ***p < 0.001) (C) Yellow arrowheads indicate CO_3_Ap granules in the CO_3_Ap and Mix groups at 6 weeks.

Micro‐CT analysis of the extraction sockets at 6 weeks post‐implantation revealed differences in bone volume/tissue volume and BMD (Figure 2A,B). The Mix and CO_3_Ap groups displayed a significantly higher bone volume compared with the AB group. In terms of BMD, both the Mix and AB groups demonstrated significantly higher values than the CO_3_Ap group, with no significant difference between the Mix and AB groups. Notably, CO_3_Ap granules were still visible in the CO_3_Ap and Mix groups at 6 weeks (Figure 2C).

### 3.3 Mucosal Tissue Closure and Structure

**FIGURE 3:**
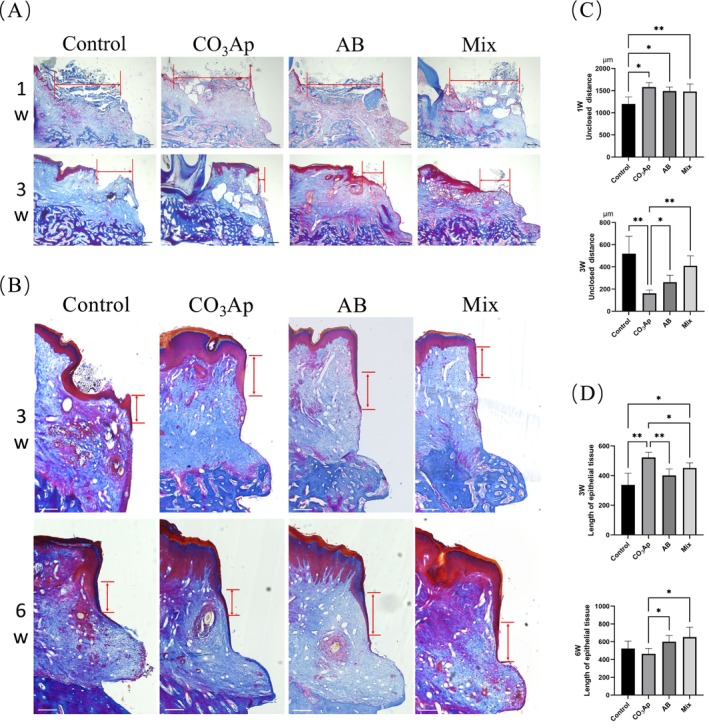
Chronological change of the mucosal tissue morphology after implantation. (A) Evaluation of mucosal closure using Ladewig's fibrin staining on epithelial unclosed samples. Bar = 200 μm. (B) Evaluation of mucosal structure using Ladewig staining on epithelial closed samples. Red arrowheads indicate the boundaries of pre‐implant epithelial tissue (PIE). Bar = 200 μm. (C) Distance between epithelial tissue and implant at 1 and 3 weeks. (D) Length of PIE at 3 and 6 weeks. (*n* = 5, *p < 0.05, **p < 0.01).

Peri‐implant soft tissue healing and epithelial attachment were evaluated using Ladewig staining (Figure 3A,B). The epithelial‐to‐implant distance was measured at 1 and 3 weeks. At 1 week, no significant differences were observed among the groups. By contrast, at 3 weeks, the CO_3_Ap group exhibited the shortest epithelial‐to‐implant distance (Figure 3C).

The pre‐implant epithelial tissue (PIE) length of mucosal closure at 3 and 6 weeks was also measured. At 3 weeks, the PIE length was significantly longer in the CO_3_Ap group compared with the other groups. By contrast, at 6 weeks, the AB and Mix groups displayed longer PIE lengths than the CO_3_Ap group (Figure 3D).

### 3.4 Osteointegration and Bone Regeneration on the Implant Surface

**FIGURE 4:**
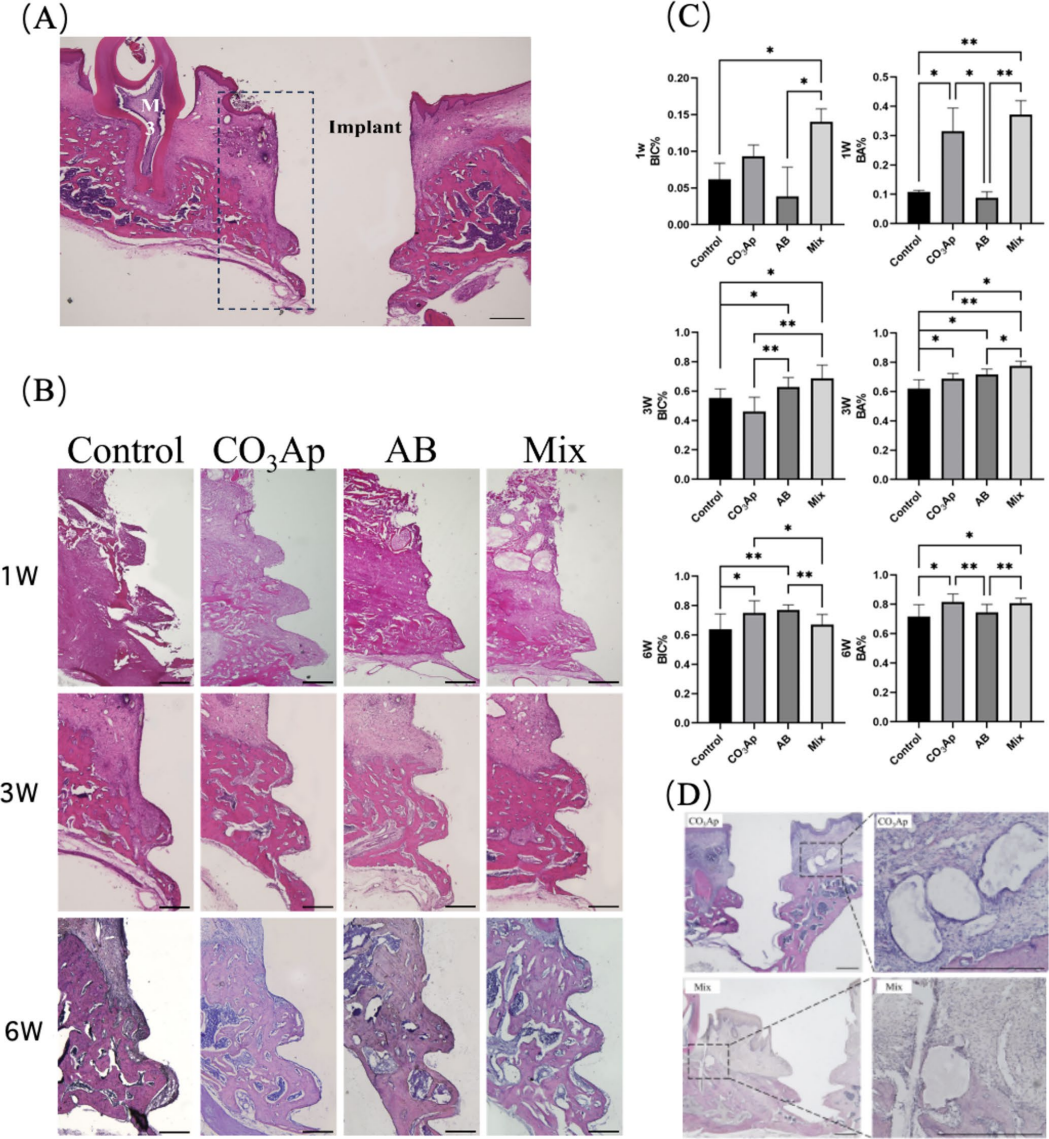
Chronological change of the bone tissue morphology after implantation. (A) Evaluation of sagittal section by hematoxylin and eosin staining. The black square indicates the observation sites. Bar = 100 μm. (B) Image of bone tissue on observation area. Bar = 500 μm. (C) Bone‐to‐implant contact ratio (BIC%) and bone area ratio (BA%) of the observation area. (*n* = 5, *p < 0.05, **p < 0.01) (D) CO_3_Ap granules in the CO_3_Ap and Mix groups at 6 weeks. Magnification reveals osteoblast‐like cells at the edge of the granules. Bar = 500 μm.

The bone‐to‐implant contact ratio (BIC%) and the peri‐implant bone area ratio (BA%) were assessed on sagittal sections, using the implant and the third molar as measurement landmarks (Figure 4A,B). At 1 week, the Mix group displayed the highest BIC% and BA%, significantly exceeding the AB group. At 3 weeks, the BIC% was higher in both the Mix and AB groups compared with the CO_3_Ap group, with no significant difference between the Mix and AB groups. The BA% remained highest in the Mix group. At 6 weeks, the BA% in the Mix and CO_3_Ap groups remained significantly higher than in the AB group (Figure 4C).

Histological observations at 6 weeks revealed the presence of residual CO_3_Ap granules in both the CO_3_Ap and Mix groups. Osteoclast‐like cells were found on the surfaces of these granules, suggesting that CO_3_Ap undergoes slow resorption and contributes to guiding new bone regeneration (Figure 4D).

### 3.5 MSC Morphology and Proliferation

**FIGURE 5:**
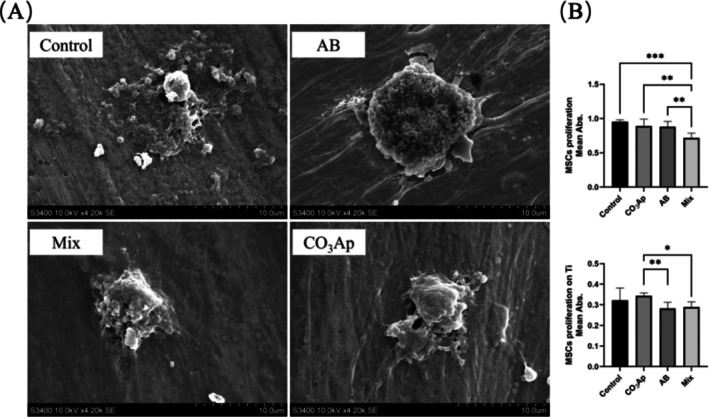
Effect of bone substitutes on MSC adhesion and proliferation. (A) Scanning electron microscopy of MSCs on the titanium plate. Magnification: ×1200. (B) Absorbance with Cell Counting Kit‐8 in culture dish and titanium plate. (*n* = 5, *p < 0.05, **p < 0.01, ***p < 0.001).

SEM analysis revealed differences in MSC morphology on titanium surfaces (Figure 5A). MSCs in the AB group were significantly larger and exhibited more protrusions toward the titanium surface compared with those in the control group. By contrast, MSCs in the CO_3_Ap group maintained smaller, more elliptical shapes.

Cell proliferation assessed using the Cell Counting Kit‐8 assay showed that on culture dishes, the Mix group exhibited the lowest MSC proliferation compared with the other groups (Figure 5B). On titanium plates, both the AB and Mix groups displayed lower cell proliferation than the CO_3_Ap group.

### 3.6 Osteogenic Differentiation of MSCs

**FIGURE 6:**
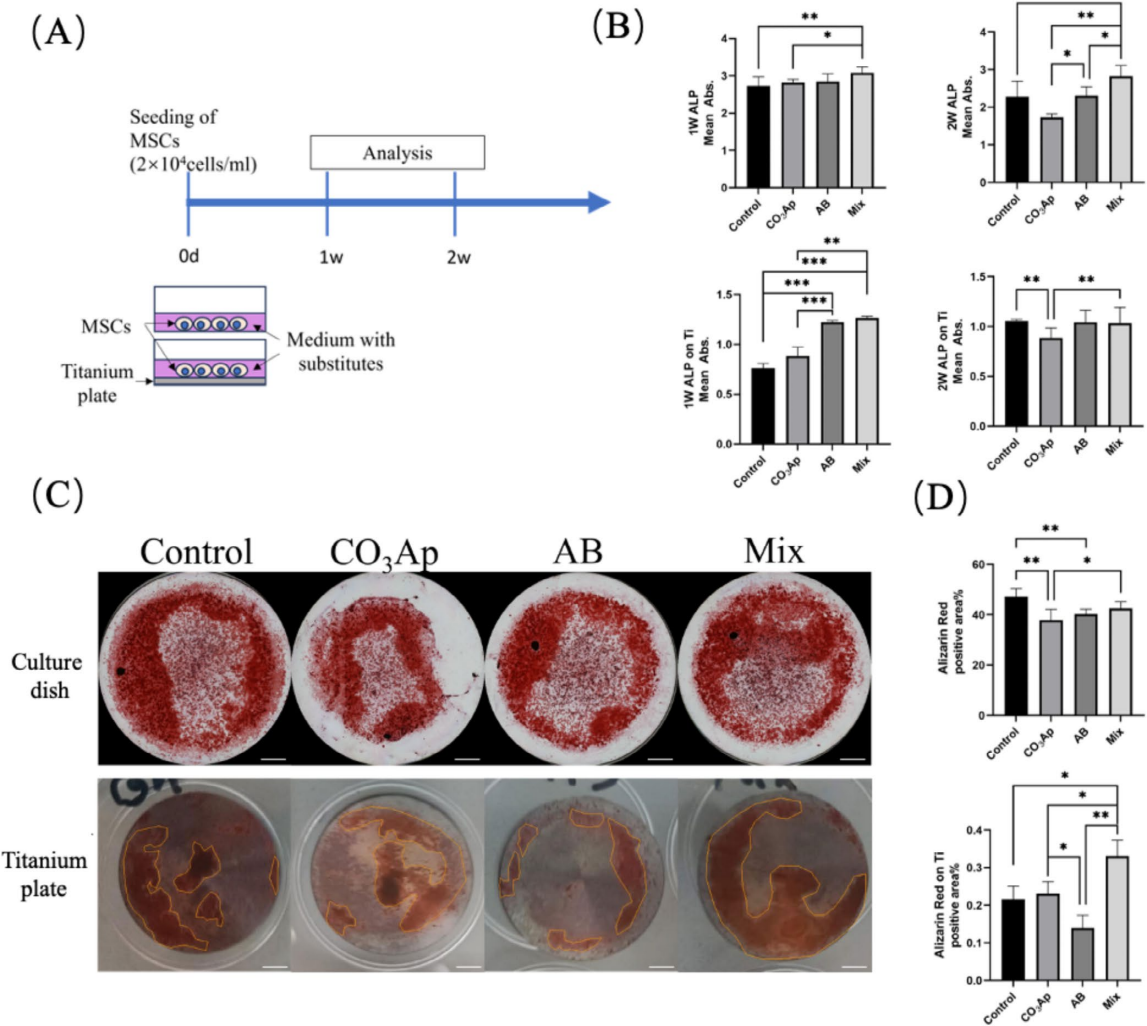
Effect of bone substitutes on MSC osteogenic differentiation. (A) Experimental protocol and schematic of the in vitro study. (B) ALP activity on culture dish or titanium plate. (C) Calcified deposits stained by Alizarin Red on culture dish and titanium plate. Bar = 2 mm. (D) Positive area of (C). (*n* = 5, *p < 0.05, **p < 0.01, ***p < 0.001).

Osteogenic differentiation was assessed through ALP activity and Alizarin Red S staining (Figure 6A). At 1 week, both the Mix and AB groups displayed significantly higher ALP activity compared with the CO_3_Ap group (Figure 6B). However, by 2 weeks, the Mix group exhibited the highest ALP activity, surpassing both the AB and CO_3_Ap groups. Alizarin Red S staining at 2 weeks revealed the most pronounced calcium nodule formation in the Mix group (Figure 6C,D).

## 4 Discussion

In this study, we evaluated the effects of CO_3_Ap, AB, and a CO_3_Ap–AB mixture on bone and soft tissue integration around titanium implants in a rat maxillary model. The results demonstrated that the CO_3_Ap–AB mixture effectively promoted both early bone regeneration and soft tissue integration, combining the advantages of both materials.

### 4.1 Early Mucosal Healing and Soft Tissue Integration

The CO_3_Ap group exhibited significantly more rapid mucosal wound closure at 3 weeks, as evidenced by visual observations and measurements of the epithelial‐to‐implant distance. These findings are consistent with previous studies indicating that CO_3_Ap promotes early wound healing because of its biocompatibility and controlled resorption characteristics [21]. The ability of CO_3_Ap to provide a stable surface while resisting inflammation likely contributed to its superior early soft tissue healing.

The PIE, a structure that aligns with the implant surface and functions similarly to the junctional epithelium in natural teeth, serves as a barrier against external stimuli and epithelial down‐growth [25, 26]. In the present study, the PIE length was used as an indicator to evaluate the structural characteristics of the peri‐implant epithelium; however, it does not directly reflect the quality or strength of epithelial attachment to the implant surface. At 6 weeks, the Mix group exhibited a longer PIE length compared with the CO_3_Ap group. While the longer PIE in the Mix group may suggest a more established epithelial structure, this observation should be interpreted cautiously, because an extended epithelial length alone does not necessarily indicate superior soft tissue integration and could potentially reflect epithelial migration rather than effective attachment.

To better understand the biological relevance of these findings, further studies are needed to evaluate the quality of epithelial attachment at the molecular level such as through the expression of adhesion proteins or integrins. The presented findings suggested that CO_3_Ap is beneficial for accelerating early mucosal healing, whereas the CO_3_Ap–AB mixture may promote a more extensive epithelial coverage, which could contribute to sealing the peri‐implant interface over time. However, the implications of these results regarding functional epithelial attachment require additional investigation.

### 4.2 Bone Regeneration and Osseointegration

In order to avoid residual material from affecting the experimental results, we treated bone materials in contact with bone as part of the regenerated bone structure, and the materials not in contact with bone were excluded from analysis [11].

Micro‐CT analysis and histological staining demonstrated that the Mix group achieved superior bone regeneration and osseointegration compared with the AB group. Specifically, bone volume in the Mix group was comparable to the CO_3_Ap group but significantly higher than the AB group, whereas BMD in the Mix group was similar to the AB group but higher than the CO_3_Ap group.

These results highlighted the synergistic effects of combining CO_3_Ap and AB. CO_3_Ap provides a scaffold for osteoclast‐mediated resorption and subsequent new bone formation [27], while AB contributes osteoinductive factors that enhance bone density and promote its maturation. The slow resorption of CO_3_Ap ensures space maintenance for new bone formation, whereas AB accelerates early bone maturation [28].

Additionally, the BIC% and BA% were significantly improved in the Mix group, particularly at 1 and 3 weeks. This suggested that the combination of CO_3_Ap and AB enhances early osseointegration, a critical factor for implant stability.

### 4.3 MSC Behavior and Osteogenic Differentiation

In vitro experiments revealed that the Mix group promoted osteogenic differentiation of MSCs while suppressing excessive cell proliferation [29, 30]. Both ALP activity and calcium nodule formation were significantly higher in the Mix group compared with the CO_3_Ap and AB groups.

The release of calcium ions [31, 32, 33] and inorganic phosphate [34] from CO_3_Ap (Ca_10‐a_(PO_4_)_6‐b_(CO_3_)_c_) activated the expression of bone‐associated proteins and promoted osteogenic differentiation of stem cells. Additionally, the osteoinductive factors present in AB likely contributed to MSC differentiation despite the slower proliferation rates [13, 35]. The combination of these two materials thus synergized their individual benefits, resulting in enhanced osteogenic potential.

Interestingly, SEM analysis revealed that MSCs in the AB group adhered more extensively to the titanium surface, indicating a strong interaction between AB particles and the implant surface [36]. However, the proliferation assay indicated that CO_3_Ap provided a more favorable environment for MSC proliferation. These results suggested a balanced interplay between osteoconductivity (CO_3_Ap) and osteoinductivity (AB) in the Mix group, leading to enhanced osteogenic potential.

### 4.4 Clinical Implications

The findings of this study suggest that a CO_3_Ap–AB mixture could serve as an effective bone substitute in dental implant procedures, particularly for patients with bone defects. The mixture combines the early wound healing properties of CO_3_Ap with the osteoinductive capacity of AB, leading to enhanced soft tissue closure, new bone formation, and osseointegration. This combination may improve implant stability and clinical success in the long term.

The slow resorption rate of CO_3_Ap ensures prolonged support during bone remodeling [37, 38], while AB accelerates early bone maturation. This balance is particularly beneficial in the oral cavity, where dynamic mechanical forces and bacterial exposure demand stable and rapid tissue integration.

### 4.5 Limitations and Future Directions

Despite the promising results, this study has several limitations. First, the rat maxillary model does not fully replicate the clinical conditions of human bone defects [39, 40]. Future studies using larger animal models or clinical trials are necessary to validate these findings in a more clinically relevant context. Second, the long‐term effects of the CO_3_Ap–AB mixture on implant stability and soft tissue maintenance were not evaluated in this study [41]. Future research should investigate the long‐term outcomes, including material degradation dynamics and the durability of the bone–soft tissue interface [42]. Finally, although the in vitro experiments provided valuable insights into MSC behavior, further molecular studies are needed to elucidate the mechanisms underlying the osteogenic effects of the CO_3_Ap–AB mixture.

## 5 Conclusion

The combination of CO_3_Ap and AB significantly promoted tissue integration, particularly early osseointegration and long‐term soft tissue sealing. By taking advantage of the osteoconductive properties of CO_3_Ap and the osteoinductive capacity of AB, this mixture demonstrates great potential as an effective bone substitute for dental implant treatments.

## Author Contributions

Conceptualization, investigation, methodology, and writing – original draft preparation: T.X. Investigation, methodology, writing – reviewing, and editing: I.A. Investigation and methodology: I.N. Investigation and data curation: B.J. Formal analysis: Y.A. Conceptualization, project: K.K. All authors have read and agreed to the published version of the manuscript.

## Conflicts of Interest

The authors declare no conflicts of interest.

## Data Availability

The data that support the findings of this study are available on request from the corresponding author. The data are not publicly available due to privacy or ethical restrictions.
